# Surgical Site Infection after Craniotomy in Neuro-Oncology (SINO): A protocol for an international prospective multicentre service evaluation across the United Kingdom and Ireland

**DOI:** 10.1371/journal.pone.0316237

**Published:** 2025-01-24

**Authors:** Keng Siang Lee, Balint Borbas, Daoud Chaudhry, Ashvin Kuri, Lawrence Best, Conor S. Gillespie, Hakim-Moulay Dehbi, Kristian Aquilina, Paul Brennan, Puneet Plaha, Keyoumars Ashkan, Michael D. Jenkinson, Stephen J. Price, British Neurosurgical Trainee Research Collaborative (BNTRC), Society of British Neurological Surgeons (SBNS), Neurology and Neurosurgery Interest Group (NANSIG)

**Affiliations:** 1 Department of Neurosurgery, King’s College Hospital, London, United Kingdom; 2 Department of Basic and Clinical Neurosciences, Maurice Wohl Clinical Neuroscience Institute, Institute of Psychiatry, Psychology and Neuroscience (IoPPN), King’s College London, London, United Kingdom; 3 Department of Neurosurgery, Queens Medical Centre, Nottingham University Hospitals NHS Trust, Nottingham, United Kingdom; 4 Department of Neurosurgery, National Hospital for Neurology and Neurosurgery, London, United Kingdom; 5 Wolfson Institute of Population Health, Queen Mary University of London, London, United Kingdom; 6 Division of Surgery and Interventional Science, Royal Free Hospital, University College London, London, United Kingdom; 7 Division of Neurosurgery, Department of Clinical Neuroscience, University of Cambridge, Addenbrooke’s Hospital, Cambridge, United Kingdom; 8 University College London Comprehensive Clinical Trials Unit, University College London, London, United Kingdom; 9 Department of Neurosurgery, Great Ormond Street Hospital for Children NHS Foundation Trust, London, United Kingdom; 10 Translational Neurosurgery, Centre for Clinical Brain Sciences, University of Edinburgh, Edinburgh, United Kingdom; 11 Department of Neurosurgery, Oxford University Hospitals NHS Foundation Trust, Oxford, United Kingdom; 12 Department of Neurosurgery, The Walton Centre NHS Foundation Trust, Liverpool, United Kingdom; 13 Institute of Systems, Molecular and Integrative Biology, University of Liverpool, Liverpool, United Kingdom; UCSF: University of California San Francisco, UNITED STATES OF AMERICA

## Abstract

**Introduction:**

Given its proximity to the central nervous system, surgical site infections (SSIs) after craniotomy (SSI-CRAN) represent a serious adverse event. SSI-CRAN are associated with substantial patient morbidity and mortality. Despite the recognition of SSI in other surgical fields, there is a paucity of evidence in the neurosurgical literature devoted to skin closure, specifically in patients with brain tumors. The primary objective of this service evaluation is to ascertain the incidence and the risk factors associated with SSI-CRAN. The secondary objectives would be a) to ascertain the incidence of SSI-CRAN in sutured versus stapled wounds, after accounting for patient, surgical and hospital confounders of SSI-CRAN and b) to determine the percentage of patients with gliomas that begin adjuvant oncological treatment in patients with infection versus those without infection.

**Methods:**

Surgical Site Infection after Craniotomy in Neuro-Oncology (SINO) is a international prospective multicentre service evaluation that will include patients with an intracranial neoplasm, both primary and secondary neoplasms, treated with cranial surgery (including biopsy). Consecutive paediatric (<18 years) and adult (≥18 years) patients diagnosed with a brain tumour, undergoing cranial surgery between 1^st^ October 2024 and 1^st^ December 2024 will be included. Prospective data will be collected with a follow-up of 90 days.

## Introduction

Given its proximity to the central nervous system, surgical site infections (SSIs) after craniotomy (SSI-CRAN) represents a serious adverse event [1]. It is associated with significant patient morbidity and mortality [2], resulting in longer length of stays (LOS) and greater hospital costs [3]. The incidence of SSI-CRAN is variable [4], averaging 4% to 5% [1, 5, 6]. Various risk factors of SSI-CRAN have been purported but these are inconsistent within the neurosurgical literature [7–10]. This inconsistency can be attributable to significant methodological heterogeneity in the current pool of evidence around the definitions of SSI and the inclusion criteria amongst primary studies [11, 12]. Additionally, as SSI-CRAN are relatively uncommon, prior studies were likely underpowered to ascertain independent predictors of SSI-CRAN [13].

A particularly large and vulnerable subset of patients undergoing craniotomy, are those with brain tumours. Patients with malignant brain tumours harbour several risk factors for SSI-CRAN such as advanced age, immunosuppression attributed to corticosteroids or chemotherapeutic agents, and local skin compromise caused by irradiation [14]. These patients therefore represents a high-risk group for SSI-CRAN. The sequelae of an SSI-CRAN in this demographic cohort could be deleterious; such as the compromise in the delivery of adjuvant oncological treatment–chemotherapy and irradiation.

The detrimental consequences of SSI-CRAN mean that identifying modifiable risk factors is highly desirable. Among the efforts to prevent the occurrence of SSI-CRAN, a variety of skin closure materials and techniques have been implemented. In both orthopaedic and obstetric surgery, the risk of developing an SSI is significantly higher when the wound is closed with staples rather than sutures [15, 16]. Despite the importance and recognition of this risk factor, there is a paucity of evidence in the neurosurgical literature devoted to skin closure. SINO will be the first prospective multi-centre service evaluation to determine the incidence of SSI-CRAN, especially in sutured versus stapled wounds based on current practices in the United Kingdom (UK) and Republic of Ireland (RI). Furthermore, knowledge of the incidence of SSI-CRAN may inform power calculations and recruitment of future interventional studies.

The primary objective of this service evaluation is to ascertain the incidence and risk factors of SSI-CRAN in neurosurgical oncology. The secondary objectives would be a) to ascertain the incidence of SSI-CRAN in sutured versus stapled wounds, after accounting for patient, surgical and hospital confounders of SSI-CRAN and b) to determine the percentage of patients with gliomas that initiate adjuvant oncological treatment in patients with infection versus those without infection.

## Materials and methods

### Outcomes

The primary outcome measure will be the incidence of SSI-CRAN, at 30 and 90 days after craniotomy. SSI-CRAN will be defined using the Centre for Disease Control (CDC) definition [17], further delineating it into superficial incisional, deep incisional and organ space infection (Table 1) [4]. The 30 and 90-day follow up periods were utilised in the CDC definition for surgeries without indwelling surgical implants.

**Table 1:** Centers for disease control and prevention definition of surgical site infection and classification of superficial, deep and organ space.

Type of SSI	Criteria
Superficial incisional SSI	Infection must occur within 30 days after any operative procedure and involve only the skin and subcutaneous tissue of incision. The patient must also have one of the following: (1) Purulent drainage from incision (2) Organisms identified from an aseptically obtained specimen (3) Superficial incision that is deliberately opened by a surgeon or other designee and culture or non- culture-based testing is not performed, and at least one of the following signs or symptoms: pain or tenderness; localized swelling; erythema; or heat. (4) Diagnosis of a superficial incisional SSI by the surgeon or an attending physician or other designee
Deep incisional SSI	Infection must occur within 30 days or 90 days after the operative procedure and involve deep soft tissues of the incision (fascial and muscle layers). The patient must also have at least one of the following: (1) Purulent drainage from the deep incision (2) A deep incision that spontaneously dehisces or is deliberately opened or aspirated by a surgeon, attending physician, or other designee, and the organism is identified by a culture or non-culture-based microbiologic testing method. The patient must also have one of the following: fever, localized pain, or tenderness. (3) An abscess or other evidence of infection involving the deep incision that is detected on gross anatomic or histopathologic examination or imaging test
Organ/space SSI	Infection occurs within 30 or 90 days after the operative procedure and involves any part of the body deeper than the fascial/muscle layers that is opened or manipulated during the operative procedure and the patient has one of the following: 1) Purulent drainage from a drain that is placed into the organ/space (2) Organisms are identified from an aseptically obtained fluid or tissue in the organ/space by a culture or non-culture-based microbiologic testing method. (3) An abscess or other evidence of infection involving the organ/space that is detected on gross anatomic or histopathologic examination or imaging test.

SSI = surgical site infection.

The secondary outcome measures will be 1) the percentage of patients which initiate postoperative adjuvant oncological treatment in the 90 day follow up 2) mortality at 30 and 90 day follow up 3) repeated operation at 30 and 90 day follow up 4) readmission at 30 and 90 day follow up and reason for readmission and 5) LOS.

### Service evaluation design

This is an international (UK and RI) multicentre prospective service evaluation, for all neurosurgical centres that perform craniotomy for intracranial neoplasms. Data will be collected on consecutive surgical patients with a confirmed diagnosis of an intracranial neoplasm, including both primary and metastatic lesions, treated with resection via craniotomy. The local collaborating team at each participating centre will prospectively collect data over a continuous period between 1^st^ October 2024 and 1^st^ December 2024.

This service evaluation is led by the Neurology and Neurosurgery Interest Group (NANSIG) [18]. This service evaluation is supported by the British Neurosurgical Trainee Research Collaborative (BNTRC) [19], academic committee of the Society for British Neurological Surgeons (SBNS) and the existing university and hospital-affiliated collaborator networks of NANSIG [20]. S1 Table provides the members of the External Advisory Group.

### Patient and centre eligibility criteria

The service evaluation will include both consecutive paediatric (<18 years) and adult (≥18 years) patients diagnosed with an intracranial neoplasm undergoing craniotomy for resection. Intracranial neoplasms will include all primary and secondary neoplasms as classified by the 2021 World Health Organisation (WHO) Classification of Tumours of the Central Nervous System [21].

Patients with no surgical intervention will be excluded. Additionally, the following patients’ index cranial surgery will be excluded to avoid heterogeneity: 1) patients who undergoing cranioplasty as part of their tumour resection, 2) endoscopic resection of tumours (e.g., patients with pituitary tumours or craniopharyngioma), 3) repeat surgeries and 4) multi-stage surgeries. A repeat surgery is defined as a craniotomy for the same tumour type, in the same anatomical location, in the same patient; due to disease progression or recurrence, such as in a recurrent glioma. However, patients who have had a previous cranial surgery (including biopsy) for a different type or location of cranial neoplasm (e.g., patient with meningioma resection and then subsequent glioma), will not be excluded.

All hospital trusts within the UK or RI with a neurosurgery service which provides neurosurgical intervention for neuro-oncological patients are eligible, including both paediatric and adult centres. All participating centres will be required to register the SINO service evaluation as per their local regulations and provide evidence of successful registration to the steering committee before the commencement of data collection.

### Patient identification and sources of information

**Fig 1:**
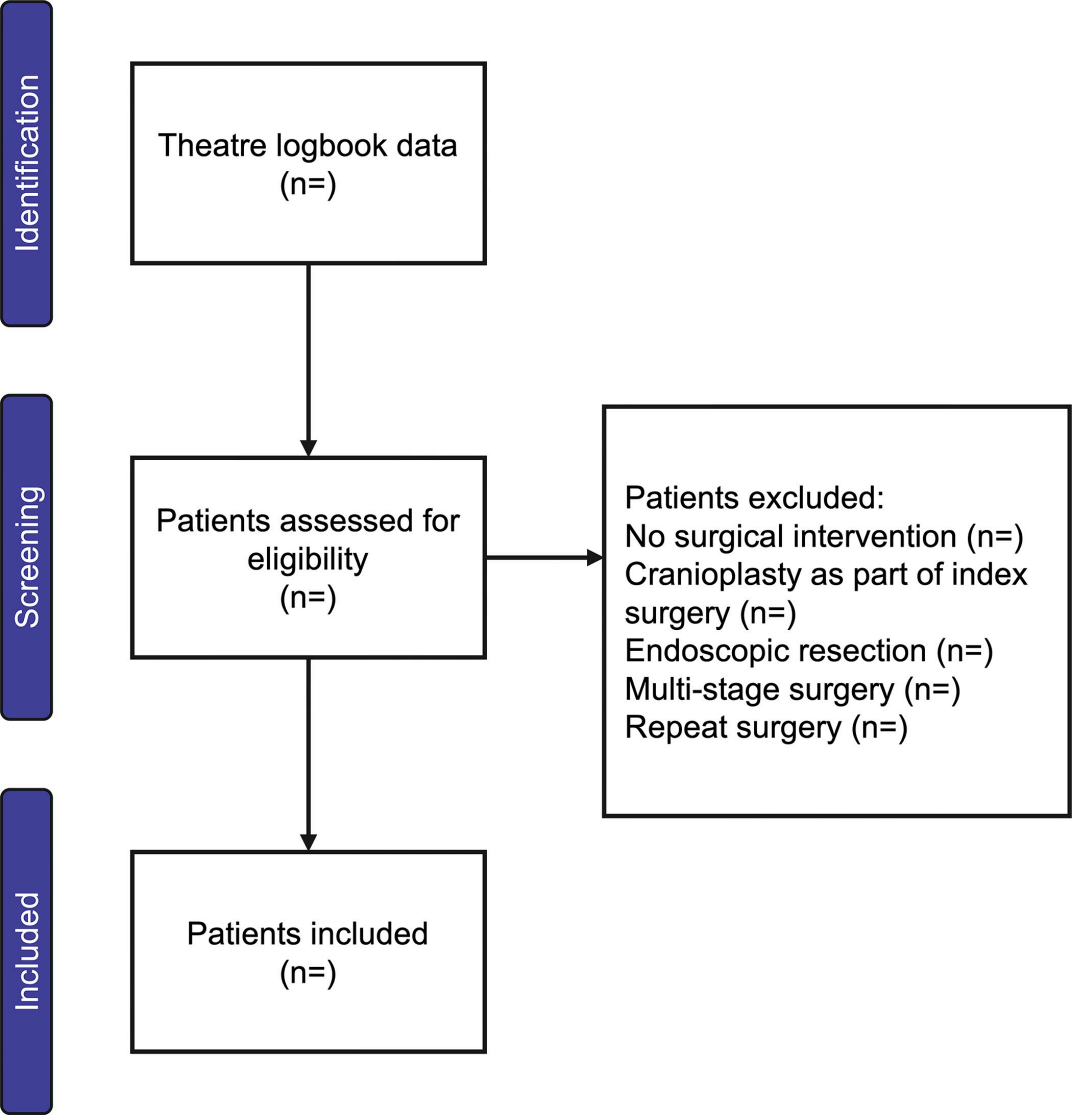
Flowchart outlining the sources of information for patient identification and eligibility.

Local collaborators will use the theatre logbooks to identify the patients, to ensure prospective patient identification. Local collaborators will assess each patient’s eligibility against the inclusion and exclusion criteria. Additional information may be gained from the surgical and neuropathology records, multidisciplinary team (MDT) documents and patients’ clinical records (Fig 1).

### Data collection

**Fig 2:**
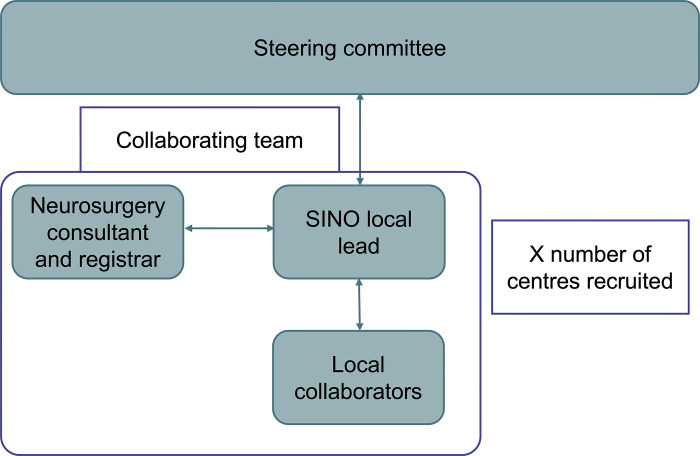
Flowchart outlining the collaboration and flow of information between each collaborating centre and the steering committee.

Each centre will have a local collaborative team formed by the SINO local lead, up to five local collaborators, a neurosurgical trainee and a supervising consultant neurosurgeon Fig 2. The collaborative team must be supported by a trainee and consultant neurosurgeon to ensure quality of data collected is maintained. Local teams are advised that if procedure notes are not clear from the operation reports, clarification should be sought from the neurosurgical trainee, and reviewed by the consultant neurosurgeon if there is still any doubt. The approach employed in this service evaluation has been previously outlined and validated. It is further detailed below [22–25]. Before commencement of data collection, all local SINO leads and local collaborators will need to attend a mandatory online training module. This comprises an introduction to SSI-CRAN and its significance, the service evaluation protocol, and a walkthrough of the bespoke Excel data collection tool. The steering committee and the data collection team will meet at one month, and at the end of the data collection phase, to address any issues.

Data will be collected at each centre by members of the local collaborating team. Data

will be collected on the demographics, comorbidities, characteristics of the neoplasm, peri-operative care, surgical details, adjuvant and neoadjuvant treatment. Data collection fields are outlined in S2 Table. The data dictionary found in S3 Table provides a complete catalogue of all data collected and associated definitions.

### Data completeness

Following data collection, only data sets with >95% data completeness will be accepted for pooled national analysis. For purposes of this service evaluation, we define data completeness as the proportion of Excel spreadsheet cells with an appropriate entry including “not reported/ not documented.” Collaborators will be withdrawn from the published list of citable collaborators if their centre does not contribute any patients, meeting the >95% data completeness level, to the service evaluation. It is important to recognise that the phrase ‘missing data’ exclusively refers to a lack of data field completion or lack of justification for failure to complete the data field. Upon submission of the Excel sheet, the steering committee will review submitted Excel sheets for any omissions or areas of concern. These will be flagged, with spreadsheets returned to be addressed by the local participating centre.

### Data validation

Data validation will be performed in all centres to audit data accuracy before data analysis.

This will involve an independent data validator who did not perform data collection in the previous submission from the centre). The steering committee will randomly select 10% of the centre’s cases, for which the assigned data validator will compare the data submitted to raw data sources for accuracy. The target for data accuracy is >90% of data items. Conflicts between actual and submitted data will be resolved by discussion between the validator and local team, with oversight from a steering committee member. If data accuracy is <90%, the local team will then be asked to update all local data accordingly. A re-audit of 10% of the centre’s cases (selected randomly) will then be repeated. If the requested updating of data is not performed or data accuracy remains <90%, data from the respective centre will be analysed separately or excluded.

### Sample size and statistical analysis

This service evaluation aims to recruit all 33 neurosurgical centres in the UK and RI. Calculations to assess the requisite sample size were done utilising the Dobson formula were performed at a 95% confidence interval, utilising a prevalence of 5% for SSI-CRAN and a requisite 0.01 precision. This yielded a sample size of 1800 to provide adequate power in order to assess incidence, which is feasible to meet in our two-month data collection window. If this requisite number of cases is not reached within this pre-specified timeframe, the collection period will be extended as necessary.

Proportions of categorical variables will be reported as counts and percentages. Normally distributed continuous data will be reported by means and their calculated standard deviations, otherwise by their median with their respective interquartile ranges. Stepwise multivariable binary logistic regression analysis will be performed to account for associated factors of SSI-CRAN and estimate the risk ratios (RRs) with corresponding 95% confidence intervals (CIs) of SSI-CRAN. Variable selection will be based on literature [7–10, 14, 26–30], and on clinically plausible variables which occur prior to the outcome event. In the univariate analyses, χ^2^ test or Fisher’s exact test will be used to compare clinical variables between infected and non-infected cases. Subgroup analyses, where applicable, will be performed for the various types of sutures (absorbable versus non-absorbable; monofilament versus braided), by histology (benign versus malignant tumors), location of the craniotomy (supratentorial versus infratentorial) and by age (paediatric versus adult cohort).

### Patient and public involvement

Public and patient involvement (PPI) was obtained for SINO and contributed to the conception of this service evaluation. Representatives from The Brain Tumour Charity provided insight and feedback on our service evaluation from the viewpoint of a carer or a patient with a brain tumour. We held a video discussion with one representative. Minutes from this meeting are in S4 Table. Two other representatives provided detailed written feedback via email. The research questions, outcome measures and data variables were developed and informed by their personal priorities, experience, and preferences. Upon discussion and reflection, it was decided to collect more data specifically targeted to determine the percentage of patients with gliomas that were unable to initiate adjuvant oncological treatment due to an SSI-CRAN. The representatives confirmed that awareness of risk factors may also help modify patient behaviours, where relevant, to limit the risk. The aim of these amendments was to enhance our service evaluation focus on patient specific concerns. The same volunteers will be involved in disseminating the results of this service evaluation through various platforms such as email mailing lists and social media.

### Ethics and dissemination

#### Service evaluation registration

This study will assess routine clinical practice without change to patient care, therefore this service evaluation requires local institutional approval in each participating centre for data collection and sharing, but does not require university or National Health Service (NHS) research ethics committee (REC) approval, as per the Health Research Authority (HRA) decision tool [31]. The service evaluation has been approved by the Nottingham University Hospitals NHS Trust hospital audit committee (approval number 24-176C). The local lead at each centre is responsible for registering this service evaluation with their respective audit departments, including Caldicott guardian and information governance approval as required. Local leads will be required to send proof of local audit and governance approvals to the service evaluation steering group prior to starting data collection.

### Local investigator responsibilities

The supervising consultant neurosurgeon of each SINO local team will be supervising the local team. It will be the responsibility of the local lead for the overall service evaluation conduct and compliance with the protocol. All the SINO local teams must have read and familiarised themselves with the protocol and the service evaluation requirements, as evidenced by attendance at the online training programme. All assisting staff (such as supervising consultants and trainees) should be informed of the protocol and its availability for review.

The SINO local lead at each centre is responsible for the quality of data recorded in the database. Supervising Consultants are responsible for assisting the SINO local team with obtaining access to relevant records prior to project start.

Each eligible centre will be responsible for establishing local data governance protocols. At UK centres, this would involve registering the service evaluation at the hospital’s clinical audit office, appointing a Caldicott Guardian to oversee data transfer flows out of the local trust. SINO will not take responsibility for local ethics approvals, however, prior to transfer of data, confirmation of appropriate registrations must be sent to the steering committee.

### Confidentiality and data protection

Patient-identifiable information such as hospital numbers will not be uploaded or stored onto the bespoke Microsoft Excel spreadsheet. Any patient-identifiable information will be collected in a separate excel sheet to ensure no accidental breach of data protection. Each new patient will be assigned a ‘Record ID’ (e.g. sequentially 1–100), on the bespoke Microsoft Excel spreadsheet. For the purposes of local traceability, each centre will collect ‘Hospital Number’ for each ‘Record ID’ in a separate excel spreadsheet. This separate excel spreadsheet contains identifiable information and is solely for internal use at the hospital centre, and will not be shared outside of the local collaborator team. This identifiable information will be maintained locally for data validation. All files containing patient data will be sorted exclusively on a password-protected hospital server or NHS protected servers. The SINO local lead will send the bespoke Microsoft Excel spreadsheet to the steering committee at sfh-tr.sino-nansig@nhs.net. All email correspondences including data will be through an nhs.net address (or local equivalent NHS protected domain) to maintain security of the data. The SINO local teams will only have access to view the records from their own centres. All data obtained should only be disclosed to the local team and the SINO steering committee. Each centre must also comply with the local trust data protection policies including requirements of the Data Protection Act 2018 and GDPR according to latest legislation.

Training on data protection is compulsory for all collaborators regardless of grade and will be provided via access to a Medical Research Council (MRC) online module. Access to overall records from every centre will be restricted to the steering committee.

Control of the complete dataset arising from this service evaluation resides with the steering committee (named in the protocol). Control of local data rests with the local audit team. Proposals to use the anonymised data should be directed to the steering committee after publication of our results. Datasets will be kept for 10 years after the conclusion of the service evaluation (this includes time to allow for analysis, and dissemination). At the end of this period, data destruction will be in line with NHS approved information destruction/deletion standards. An IT supplier certificate of destruction will be obtained.

## Discussion

### Limitations of study design

The service evaluation results may not be fully generalisable to studies beyond the UK and RI, however is intended to be the largest prospective UK and RI study on the topic. The use of a mandatory training module will enable standardised data collection, with clarity of definitions and instructions. The prospective design may limit case numbers and subsequently statistical power, but is useful for establishing a temporal relationship and assessing incidence whilst minimising selection bias. Eventually, our results will be reported in accordance with the Strengthening the Reporting of Observational studies in Epidemiology (STROBE) guidelines.

## Supporting information

S1 Checklist STROBE statement—checklist of items that should be included in reports of *cohort studies*. (DOCX)

S1 Table Surgical Site Infection after Craniotomy in Neuro-Oncology (SINO): External advisory group. Logo was reprinted from Keng Siang Lee under a CC BY license, with permission from Keng Siang Lee, original copyright 2024.
(DOCX)

S2 Table Data extraction proforma. (DOCX)

S3 Table Data dictionary. (DOCX)

S4 Table Minutes from public and patient involvement (PPI) meeting. (DOCX)
