# Correction: Surgical Site Infection after Craniotomy in Neuro-Oncology (SINO): A protocol for an international prospective multicentre service evaluation across the United Kingdom and Ireland

**DOI:** 10.1371/journal.pone.0324190

**Published:** 2025-05-14

**Authors:** Keng Siang Lee, Balint Borbas, Daoud Chaudhry, Ashvin Kuri, Lawrence Best, Conor S. Gillespie, Hakim-Moulay Dehbi, Kristian Aquilina, Paul Brennan, Puneet Plaha, Keyoumars Ashkan, Michael D. Jenkinson, Stephen J. Price, Neurology and Neurosurgery Interest Group (NANSIG)

The British Neurosurgical Trainee Research Collaborative (BNTRC) and Society of British Neurological Surgeons (SBNS) should not have been included in the author byline.

## References

[1] Lee KS, Borbas B, Chaudhry D, Kuri A, Best L, Gillespie CS, et al. Surgical Site Infection after Craniotomy in Neuro-Oncology (SINO): A protocol for an international prospective multicentre service evaluation across the United Kingdom and Ireland. PLoS One. 2025;20(1): e0316237. doi: 10.1371/journal.pone.0316237 39854365 PMC11759407

