# Supplementary material for: Surgical Site Infection after Craniotomy in Neuro-Oncology (SINO): A protocol for an international prospective multicentre service evaluation across the United Kingdom and Ireland
Source: PLoS One. 2025 Jan 24;20(1):e0316237. doi: 10.1371/journal.pone.0316237 (PMC11759407; doi:10.1371/journal.pone.0316237)
Supplement: S1 Table — Logo was reprinted from Keng Siang Lee under a CC BY license, with permission from Keng Siang Lee, original copyright 2024. (DOCX) [file pone.0316237.s002.docx]

**S1 Table 1. Surgical Site Infection After Craniotomy in Neuro-Oncology (SINO): External Advisory Group**

Kiran Cheema – The Brain Tumour Charity, Hampshire, UK

David Henshall – Department of Neurosurgery, Royal Infirmary of Edinburgh, Edinburgh, UK

Yousif Ali – University College London Medical School, University College London, United Kingdom

Setthasorn Oi – Cardiff University School of Medicine, Cardiff, UK

Mariyam Mujeeb – Department of Neurosurgery, Queens Medical Centre, Nottingham University Hospitals NHS Trust, Nottingham, UK

Omar Kouli – Department of Neurosurgery, Queen Elizabeth Hospital, Birmingham, UK

Abdurrahman I Islim – Department of Neurosurgery, The Walton Centre NHS Foundation Trust, Liverpool, UK

Sara Venturini – Division of Neurosurgery, Department of Clinical Neuroscience, University of Cambridge, Addenbrooke's Hospital, Cambridge, UK

Aswin Chari – Department of Neurosurgery, Great Ormond Street Hospital for Children NHS Foundation Trust, London, UK

Sheikh MB Momin – Department of Neurosurgery, Queen Elizabeth Hospital, Birmingham, UK

Emily R Bligh – Department of Neurosurgery, Wessex Neurological Centre, University Hospital Southampton NHS Foundation Trust, Southampton, UK

Ellie Edlmann – Division of Neurosurgery, Department of Clinical Neuroscience, University of Cambridge, Addenbrooke's Hospital, Cambridge, UK

Jumoke Sule – Clinical Microbiology and Public Health Laboratory, Health Protection Agency, Addenbrooke's Hospital, Cambridge, UK
