# Supplementary material for: Surgical Site Infection after Craniotomy in Neuro-Oncology (SINO): A protocol for an international prospective multicentre service evaluation across the United Kingdom and Ireland
Source: PLoS One. 2025 Jan 24;20(1):e0316237. doi: 10.1371/journal.pone.0316237 (PMC11759407; doi:10.1371/journal.pone.0316237)
Supplement: S2 Table — (DOCX) [file pone.0316237.s003.docx]

**S2 Table.2. Data extraction proforma**

| Patient demographics | | | | | | | | |  |
| --- | --- | --- | --- | --- | --- | --- | --- | --- | --- |
| Hospital  code | patient  record | Record ID | Sex at  birth | Age at  surgery | age in months if <2 yeas | BMI | Index of multiple deprivation | Ethnicity |  |
|  |  |  |  |  |  |  |  |  |  |

| Comorbidities | | | | | | | | | | | | | | | | | | | | | | | |  |
| --- | --- | --- | --- | --- | --- | --- | --- | --- | --- | --- | --- | --- | --- | --- | --- | --- | --- | --- | --- | --- | --- | --- | --- | --- |
| Smoking status | ASA grade | MI | CHF | peripheral  vascular  disease | CVA/TIA | Neurological  deficit | dementia | COPD | connective tissue  disease | liver disease | diabetes | CKD | solid  tumour | leukaemia | lymphoma | AIDS | hypertension | hyperlipidaemia | pre-operative immunosupressive medication | Date of last dose of immunosuppressive medication | neo-adjuvant chemotherapy | neo-adjuvant radiotherapy | Free text comorbidities | |

| Details of previous cranial surgery /SSI | | | | | | | | | | | | | | | | | | |
| --- | --- | --- | --- | --- | --- | --- | --- | --- | --- | --- | --- | --- | --- | --- | --- | --- | --- | --- |
| previous  craniotomy | if Yes, date  of SSI-CRAN | | | Indication for previous craniotomy | location of  previous craniotomy | laterality | previous cranial biopsy for  present neoplasm | if Yes, date  of SSI-CRAN | | | location of biopsy | laterality | previous  SSI-CRAN | if Yes, date  of SSI-CRAN | | | location of  previous SSI-CRAN | laterality |
|  | DD | MM | YYYY |  |  |  |  | DD | MM | YYYY |  |  |  | DD | MM | YYYY |  |  |

| peri-operative data | | | | | | | | | | | | | | | | | | | | | | | | | | | | | | | | | | | |  |
| --- | --- | --- | --- | --- | --- | --- | --- | --- | --- | --- | --- | --- | --- | --- | --- | --- | --- | --- | --- | --- | --- | --- | --- | --- | --- | --- | --- | --- | --- | --- | --- | --- | --- | --- | --- | --- |
| anaesthetic  type | FiO2 | Preoperative HbA1C | Preoperative capillary blood glucose | peri-op antibiotics prophylaxis | number of agents given | Agent 1 | Dose | frequency | duration | Agent 2 | Dose | frequency | duration | Agent 3 | Dose | frequency | duration | other cause for antibiotics given | pre-op steroid  use | steroid  type | steroid dose  (mg/day) | Length of pre-op steroid course | weaned to stop | weaned to maintenance | continued maintenance dose (mg/day) | post-op steroid  use | steroid  type | steroid dose  (mg/day) | Length of post-op steroid course | weaned to stop | weaned to maintenance | continued maintenance dose (mg/day) | prior cranial  radiation | number of fractions of radiation | dose of radiation (Grays) |  |
|  |  |  |  |  |  |  |  |  |  |  |  |  |  |  |  |  |  |  |  |  |  |  |  |  |  |  |  |  |  |  |  |  |  |  |  |  |

| Surgical details | | | | | | | | | | | | | | | | | | | | |
| --- | --- | --- | --- | --- | --- | --- | --- | --- | --- | --- | --- | --- | --- | --- | --- | --- | --- | --- | --- | --- |
| Date of  surgery | | | surgical  prep | Length of  operation (min) | Cranial location | laterality | Tumour Classification | Tumour size (mm) | suture  type | suture material | absorbable? | staples | articifical dura | extent of  resection | grade of  lead surgeon | EVD insertion | Wound drain insertion | Changing of gloves/ instruments | intent of  surgery |  |
| DD | MM | YYYY |  |  |  |  |  |  |  |  |  |  |  |  |  |  |  |  |  |  |

| Outcomes | | | | | | | | | | | | | | | | | | | | | | |
| --- | --- | --- | --- | --- | --- | --- | --- | --- | --- | --- | --- | --- | --- | --- | --- | --- | --- | --- | --- | --- | --- | --- |
| date of admission | | | | date of discharge | | | | LOS | readmission in 30 days | readmission reason | SSI- CRAN  (D30) | date of  SSI | | | | delay to  post-operative adjuvant therapy | failure to proceed with post-operative adjuvant therapy | Clavein Dindo Classification | repeat  operation | indication for re-op | 30-day  mortality | Cause of  Mortality |
| DD | MM | YY | DD/ MM/ YY | DD | MM | YY | DD/ MM/ YY |  |  |  |  | DD | MM | YY | DD/ MM/ YY |  |  |  |  |  |  |  |
