# Supplementary material for: Surgical Site Infection after Craniotomy in Neuro-Oncology (SINO): A protocol for an international prospective multicentre service evaluation across the United Kingdom and Ireland
Source: PLoS One. 2025 Jan 24;20(1):e0316237. doi: 10.1371/journal.pone.0316237 (PMC11759407; doi:10.1371/journal.pone.0316237)
Supplement: S3 Table — (DOCX) [file pone.0316237.s004.docx]

**S3 Table 3. Data dictionary**

| **Data Item** | **Definition** | **Data options** |
| --- | --- | --- |
| **Patient demographics** | | |
| Sex | Sex at birth | Male/ Female |
| Age | Age at time of surgery | Free text- number |
| Age | age in months if <2 years | Free text- number  N/A |
| BMI | Body mass index | Free text- number  Not documented |
| Index of multiple deprivation | Index of multiple deprivation based on UK data, reported in deciles. <https://data.cdrc.ac.uk/dataset/index-multiple-deprivation-imd#:~:text=The%20Index%20of%20Multiple%20Deprivation,on%20its%20own%20data%20portal>. | Numbers 1 to 10, where 10 is the least deprived |
| Ethnicity | As according to standard NHS ethnic code.  NHS Data Model and Dictionary: [ETHNIC CATEGORY](https://www.datadictionary.nhs.uk/data_elements/ethnic_category.html) | White – British  White – Irish  White – Any other White background  Mixed – White and Black Caribbean  Mixed – White and Black African  Mixed – White and Asian  Mixed – Any other mixed background  Asian or Asian British – Indian  Asian or Asian British – Pakistani  Asian or Asian British – Bangladeshi  Asian or Asian British – Any other Asian background  Black or Black British – Caribbean  Black or Black British – African  Black or Black British – Any other Black background  Other Ethnic Groups – Chinese  Other Ethnic Groups – Any other ethnic group  Not documented |
| **Comorbidities** | | |
| Smoking status | Smoking status recorded in patients’ medical notes. | Not reported  Current smoker  Ex/previous smoker  Never smoker  Non-smoker (not specified whether ex or never)  Not documented |
| ASA grade | American Society of Anaesthesiologist recorded in surgical notes | I: Normal healthy patient  II: Patient with mild systemic disease  III: Patient with severe systemic disease  IV: Patient with severe systemic disease that is a constant threat to life  V: Moribund patient not expected to survive without the operation  Not documented |
| Myocardial infarction | Previous myocardial infarction | Yes  No  Not documented |
| Congestive heart failure | Current congestive heart failure | Yes  No  Not documented |
| Peripheral vascular disease | Current peripheral vascular disease | Yes  No  Not documented |
| CVA/TIA | Previous Cerebral vascular accident/Transient Ischaemic Attack | Yes  No  Not documented |
| Neurological deficit | Neurological deficit due to previous CVA | Free text  N/A |
| Dementia | Diagnosed dementia | Yes  No  Not documented |
| COPD | Diagnosed Chronic Obstructive Pulmonary Disease | Yes  No  Not documented |
| Connective Tissue Disease | Diagnosed connective Tissue Disease | Yes  No  Not documented |
| Liver disease | Diagnosed liver disease. **Severe** = cirrhosis and portal hypertension with variceal bleeding history  **Moderate** = cirrhosis and portal hypertension but no variceal bleeding history  M**ild** = chronic hepatitis (or cirrhosis without portal hypertension) | None  Mild  Moderate  Severe  Not documented |
| Diabetes | Diagnosed diabetes mellitus | None  Pre-diabetic  T1DM  T2DM-- Diet controlled  T2DM- Oral anti-diabetic controlled  T2DM- Insulin dependent  Not documented |
| Chronic kidney disease | **Severe** = on dialysis, status post kidney transplant, uraemia  **Moderate** = creatinine >3 mg/dL (0.27 mmol/L) | CKD1  CKD2  CKD3A  CKD3B  CKD4  CKD5  Not documented |
| Solid Tumour | Diagnosis of any none neurological solid tumour | None  Localised  Metastatic  Not documented |
| Leukaemia | Diagnosis of leukaemia | Yes  No  Not documented |
| Lymphoma | Diagnosis of lymphoma | Yes  No  Not documented |
| AIDS | Diagnosis of AIDS | Yes  No  Not documented |
| Hypertension | Diagnosis of hypertension | Yes  No  Not documented |
| Hyperlipidaemia | Diagnosis of hyperlipidaemia | Yes  No  Not Documented |
| Pre-operative immunosuppressive medication | Use of immunosuppressive medication within 7 days of surgery | Yes  No  Not documented |
| Date of last dose of immunosuppressive medication | Date of last dose of immunosuppressive medication if within the 7 day window. | DD/MM/YYYY  N/A  Not documented |
| Neoadjuvant chemotherapy | Use of neoadjuvant chemotherapy | Yes  No  Not documented |
| Neoadjuvant radiotherapy | Use of neoadjuvant radiotherapy | Yes  No  Not documented |
| Previous Cranial surgery/SSI | | |
| Previous Cranial surgery | Previous cranial surgery for a different indication or in a different cranial location | Yes  No  Not documented |
| Date of previous cranial surgery | If previous cranial surgery, date of this surgery | DD/MM/YYYY  N/A  Not documented |
| Indication for previous cranial surgery | Reason for previous cranial surgery. | Vascular  Oncological  Trauma  Not documented |
| Location of  previous cranial surgery | Location of cranial neoplasm. | Temporal  Frontal  Parietal  Occipital  Infratentorial  Not documented |
| Laterality | Side of the cranium neoplasm is located | Left  Right  Bilateral  Not documented |
| Cranial biopsy for present neoplasm | Was a transcranial biopsy performed prior to the index surgery for this neoplasm. | Yes  No  Not documented |
| Date of biopsy for present neoplasm | If biopsy was performed, the date of the biopsy | DD/MM/YYYY  N/A  Not documented |
| Location of biopsy | Location of biopsy entry point. | Temporal  Frontal  Parietal  Occipital  Infratentorial  Not documented |
| Laterality | Side of the cranium of biopsy entry point. | Left  Right  Not documented |
| Previous SSI-CRAN | If previous cranial surgery, occurrence of previous SSI-CRAN. | Yes  No  Not documented |
| Date of previous SSI-CRAN | If previous SSI-CRAN, date of SSI-CRAN | DD/MM/YYYY  N/A  Not documented |
| Location of previous SSI-CRAN | Location of previous SSI-CRAn | Temporal  Frontal  Parietal  Occipital  Infratentorial  Not documented |
| Laterality | Side of the cranium of previous SSI-CRAN | Left  Right  Bilateral  Not documented |
| **Perioperative Data** | | |
| Anaesthetic  type | Anaesthetic used during cranial surgery | General  Awake  Not documented |
| FiO2 | Fraction of inspired oxygen during surgery | percentage  Not documented |
| Preoperative HbA1C | Preoperative HbA1C, within the last 3 months. Recorded in mmol/mol | Number  Not documented |
| Preoperative capillary blood glucose | Preoperative capillary blood glucose, within the last 24 hours months. Recorded in mmol/L | Number  Not documented |
| Perioperative antibiotics prophylaxis | Use of antibiotics prior to, during or after surgery | Yes (pre-operative)  Yes (intra-operative)  Yes (post-operative)  Yes (a combination of pre, intra or post-operative)  No  Not documented |
| Number of agents given | Number of antibiotics used in the perioperative period | Free text – number  Not documented |
| Agent 1 | First antibiotic used | Free text  N/A |
| Dose | Dose of antibiotic given in milligrams | Free text – number  N/A |
| Frequency | Frequency of antibiotic administration | Once only (OO)  Once daily (OD)  Twice daily (BD)  Three times daily (TDS)  Four times daily (QDS)  Other  Not documented |
| Duration | Duration of antibiotic course in days. (if once only then N/A) | Free text – number  N/A |
| Agent 2 | Second antibiotic used | Free text  N/A |
| Dose | Dose of antibiotic given in milligrams | Free text – number  N/A |
| Frequency | Frequency of antibiotic administration | Once only (OO)  Once daily (OD)  Twice daily (BD)  Three times daily (TDS)  Four times daily (QDS)  Other  Not documented |
| Duration | Duration of antibiotic course in days. (if once only then N/A) | Free text – number  N/A |
| Agent 3 | Third antibiotic used | Free text  N/A |
| Dose | Dose of antibiotic given in milligrams | Free text – number  N/A |
| Frequency | Frequency of antibiotic administration | Once only (OO)  Once daily (OD)  Twice daily (BD)  Three times daily (TDS)  Four times daily (QDS)  Other  Not documented |
| Duration | Duration of antibiotic course in days. (if once only then N/A) | Free text – number  N/A |
| Cause for antibiotic use | Why were the antibiotics being prescribed to the patient | Prophylaxis  Treatment- SSI  Treatment- systemic infection  Not documented |
| Preoperative steroid  use | Use of systemic corticosteroids prior to surgery | Yes  No  Not documented |
| Steroid   type | Name and route of administration of corticosteroid | Free text  N/A |
| Steroid dose  (mg/day) | Dose of corticosteroid per day | Free text – number  N/A |
| Length of steroid course | Number of days taking corticosteroids | Free text - number  N/A |
| Steroid weaned to stop | Corticosteroid dose weaned to stop. | Yes  No  Not documented |
| Steroid weaned to maintenance | Corticosteroid dose weaned to a maintenance dose. | Yes  No  Not documented |
| Continued maintenance dose | Dose of maintenance corticosteroid given | Free text - number  N/A |
| Post-operative steroid use | Use of systemic corticosteroids after the surgery | Yes  No  Not documented |
| Steroid   type | Name and route of administration of corticosteroid | Free text  N/A |
| Steroid dose  (mg/day) | Dose of corticosteroid in mg/day | Free text – number  N/A |
| Length of steroid course | Number of days taking corticosteroids | Free text - number  N/A |
| Steroid weaned to stop | Corticosteroid dose weaned to stop. | Yes  No  Not documented |
| Steroid weaned to maintenance | Corticosteroid dose weaned to a maintenance dose. | Yes  No  Not documented |
| Continued maintenance dose (mg/day) | Dose of maintenance corticosteroid given in mg/day | Free text - number  N/A |
| Prior cranial  radiation | Previous exposure to cranial radiotherapy | Yes  No  Not documented |
| Number of fractions of radiation | Number of fractions of cranial radiotherapy used | Free text – number  N/A |
| Dose of radiation (Grays) | Dose of previous exposure to cranial radiotherapy in Grays | Free text - number  N/A |
| **Surgical Details** | | |
| Date of  surgery | Date in DD/MM/YYYY | DD/MM/YYYY |
| Surgical  preparation | Type of surgical preparation solution used prior to surgery | Iodine  Chlorhexidine  Both  Not documented |
| Length of  operation (min) | Length of surgery in minutes, from incision to closure. | Free text – number  Not documented |
| Cranial location | Location of cranial tumour. | Temporal  Frontal  Parietal  Occipital  Infratentorial  Not documented |
| Laterality | Side of the cranium tumour is located | Left  Right  Not documented |
| Tumour classification | Tumour classification based on 2021 WHO Classification of Tumours of the Central Nervous System | **Gliomas, glioneuronal tumours, and neuronal tumours**   Adult-type diffuse gliomas    Astrocytoma, IDH-mutant    Oligodendroglioma, IDH-mutant, and 1p/19q-codeleted    Glioblastoma, IDH-wildtype   Paediatric-type diffuse low-grade gliomas    Diffuse astrocytoma, MYB- or MYBL1-altered    Angiocentric glioma    Polymorphous low-grade neuroepithelial tumour of the young    Diffuse low-grade glioma, MAPK pathway-altered   Paediatric-type diffuse high-grade gliomas    Diffuse midline glioma, H3 K27-altered    Diffuse hemispheric glioma, H3 G34-mutant    Diffuse paediatric-type high-grade glioma, H3-wildtype and IDH-wildtype    Infant-type hemispheric glioma   Circumscribed astrocytic gliomas    Pilocytic astrocytoma    High-grade astrocytoma with piloid features    Pleomorphic xanthoastrocytoma    Subependymal giant cell astrocytoma    Chordoid glioma    Astroblastoma, MN1-altered   Glioneuronal and neuronal tumours    Ganglioglioma    Desmoplastic infantile ganglioglioma / desmoplastic infantile astrocytoma    Dysembryoplastic neuroepithelial tumour    Diffuse glioneuronal tumour with oligodendroglioma-like features and nuclear clusters    Papillary glioneuronal tumour    Rosette-forming glioneuronal tumour    Myxoid glioneuronal tumour    Diffuse leptomeningeal glioneuronal tumour    Gangliocytoma    Multinodular and vacuolating neuronal tumour    Dysplastic cerebellar gangliocytoma (Lhermitte-Duclos disease)    Central neurocytoma    Extraventricular neurocytoma    Cerebellar liponeurocytoma   Ependymal tumours    Supratentorial ependymoma    Supratentorial ependymoma, ZFTA fusion-positive    Supratentorial ependymoma, YAP1 fusion-positive    Posterior fossa ependymoma    Posterior fossa ependymoma, group PFA    Posterior fossa ependymoma, group PFB    Spinal ependymoma    Spinal ependymoma, MYCN-amplified    Myxopapillary ependymoma    Subependymoma  **Choroid plexus tumours**   Choroid plexus papilloma   Atypical choroid plexus papilloma   Choroid plexus carcinoma  **Embryonal tumours**   Medulloblastoma    Medulloblastomas, molecularly defined     Medulloblastoma, WNT-activated     Medulloblastoma, SHH-activated and TP53-wildtype     Medulloblastoma, SHH-activated and TP53-mutant     Medulloblastoma, non-WNT/non-SHH    Medulloblastomas, histologically defined   Other CNS embryonal tumours    Atypical teratoid/rhabdoid tumour    Cribriform neuroepithelial tumour    Embryonal tumour with multilayered rosettes    CNS neuroblastoma, FOXR2-activated    CNS tumour with BCOR internal tandem duplication    CNS embryonal tumour  Pineal tumours   Pineocytoma   Pineal parenchymal tumour of intermediate differentiation   Pineoblastoma   Papillary tumour of the pineal region   Desmoplastic myxoid tumour of the pineal region, SMARCB1-mutant  **Cranial and paraspinal nerve tumours**   Schwannoma   Neurofibroma   Perineurioma   Hybrid nerve sheath tumour   Malignant melanotic nerve sheath tumour   Malignant peripheral nerve sheath tumour   Paraganglioma  **Meningiomas**   Meningioma  **Mesenchymal, non-meningothelial tumours**   Soft tissue tumours    Fibroblastic and myofibroblastic tumours     Solitary fibrous tumour    Vascular tumours     Hemangiomas and vascular malformations     Hemangioblastoma    Skeletal muscle tumours     Rhabdomyosarcoma    Uncertain differentiation     Intracranial mesenchymal tumour, FET-CREB fusion-positive     CIC-rearranged sarcoma     Primary intracranial sarcoma, DICER1-mutant     Ewing sarcoma   Chondro-osseous tumours    Chondrogenic tumours     Mesenchymal chondrosarcoma     Chondrosarcoma    Notochordal tumours     Chordoma (including poorly differentiated chordoma)  **Melanocytic tumours**   Diffuse meningeal melanocytic neoplasms    Meningeal melanocytosis and meningeal melanomatosis   Circumscribed meningeal melanocytic neoplasms    Meningeal melanocytoma and meningeal melanoma  **Hematolymphoid tumours**   Lymphomas    CNS lymphomas     Primary diffuse large B-cell lymphoma of the CNS     Immunodeficiency-associated CNS lymphoma     Lymphomatoid granulomatosis     Intravascular large B-cell lymphoma    Miscellaneous rare lymphomas in the CNS     MALT lymphoma of the dura     Other low-grade B-cell lymphomas of the CNS     Anaplastic large cell lymphoma (ALK+/ALK−)     T-cell and NK/T-cell lymphomas   Histiocytic tumours    Erdheim-Chester disease    Rosai-Dorfman disease    Juvenile xanthogranuloma    Langerhans cell histiocytosis    Histiocytic sarcoma  **Germ cell tumours**   Mature teratoma   Immature teratoma   Teratoma with somatic-type malignancy   Germinoma   Embryonal carcinoma   Yolk sac tumour   Choriocarcinoma   Mixed germ cell tumour  **Metastases to the CNS**   Metastases to the brain and spinal cord parenchyma   Metastases to the meninges |
| Tumour size (mm) | Dimensions of the tumour x*y*z recorded in mm | Free text – number  Not documented |
| Suture  type | Whether the suture used in skin closure is braided or monofilament. | Braided  Monofilament  Not documented |
| Suture material | The material of the suture used in skin closure | Vicryl  Monocryl  PDS  Silk  Polyester  Nylon  Polypropylene  Not documented |
| Absorbable? | Whether the suture used in skin closure is absorbable or non-absorbable. | Absorbable  Non-absorbable  Not documented |
| Staples | Use of staples in skin closure. | Yes  No  Not documented |
| Artificial dura | Use of artificial dura in closure. | Yes  No  Not documented |
| Extent of  resection | Extent of resection documented in surgical record | Total  Subtotal  Not documented |
| Grade of  lead surgeon | Grade of most senior surgeon scrubbed for the procedure. | Consultant  Registrar  Post-CCT  Not documented |
| EVD insertion | Insertion of an EVD during index surgery | Yes  No  Not documented |
| Wound drain insertion | Insertion of an wound drain during index surgery | Yes  No  Not documented |
| Changing of gloves/ instruments | Changing of gloves/ instruments during the closure in the index surgery | Yes  No  Not documented |
| Intent of  surgery | Intention of the surgery | Palliative  Curative  Not documented |
| **Outcomes** | | |
| length of  stay (days) | Length of stay post-surgery in hospital. In days. | Free text-  number  Not documented |
| Date of admission | Date in DD/MM/YYYY | DD/MM/YYYY  Not documented |
| Date of discharge | Date in DD/MM/YYYY | DD/MM/YYYY  Not documented |
| Readmission | Was the patient readmitted within 30 days of index surgery | Yes  No  Not documented |
| Readmission reason | Cause of readmission | Free text  N/A |
| SSI-CRAN (D30) | Occurrence of SSI-CRAN within 30 days. | Yes- superficial  Yes- deep  Yes- organ space  No  Not documented |
| Date of  SSI-CRAN | Date in DD/MM/YYYY | DD/MM/YYYY  N/A |
| Delay to  post-operative adjuvant therapy |  | Yes  No |
| Failure to proceed with post-operative adjuvant therapy |  | Yes  No |
| Clavien Dindo Classification | Clavien Dindo Classification of surgical complications defined as in Figure 3 (https://www.ncbi.nlm.nih.gov/pmc/articles/PMC1360123/) | 1  2  3a  3b  4a  4b  5  none  not documented |
| Repeat  operation | Repeat cranial surgery | Yes  No  Not documented |
| Indication for reoperation |  | Free text  N/A |
| Mortality (D30) | Mortality of any cause at 30 days post-index surgery | Yes  No  Not documented |
| Cause of mortality | Cause of mortality | Free text  N/A |
