# Supplementary material for: Surgical Site Infection after Craniotomy in Neuro-Oncology (SINO): A protocol for an international prospective multicentre service evaluation across the United Kingdom and Ireland
Source: PLoS One. 2025 Jan 24;20(1):e0316237. doi: 10.1371/journal.pone.0316237 (PMC11759407; doi:10.1371/journal.pone.0316237)
Supplement: S4 Table — (DOCX) [file pone.0316237.s005.docx]

**S4 Table 4. Minutes from Public and patient involvement (PPI) meeting**

| **Date: 17/11/2023** |
| --- |
| **Time: 11:00**  Meeting with NC a patient who is remission after an intracranial neoplasm.  **Question**  Do you feel that this service evaluation is worthwhile?  **Answer**  Yes, knowing that all consideration which may reduce the likelihood of infection would be useful in her opinion both for the patients and their families.  **Question**  How clear was the information disseminated to yourself regarding the service evaluation?  **Answer**  The information was clear, but she feels that she would like additional information regarding the service evaluation, specifically on how this would affect care, are any additional people going to be present in theatre.  **Question**  Do you think this service evaluation would be useful to patients with intracranial neoplasms?  **Answer**  Yes, she thinks this will help with patient, family and carer anxiety around the surgery and risk of infection. Additionally, may help to alleviate COVID-19 concerns. She believes the information yielded from this service evaluation would help her to feel better regarding the surgery and benefit her recovery.  **Question**  How much do you think patients wish to know regarding infection risk?  **Answer**  She believes this is personal for each patient, but for herself she would like to know as much as possible, as she had a difficult diagnosis. However, she comments that others may not wish to have as much detail.  **Question**  How best to disseminate the findings to patients who are interested?  **Answer**  Via small and digestible hand-outs, ideally paper forms as she feels that it is more authoritative which could help put patients at ease. Alternatives may be via email or online forms.  **Question**  Would she like to know about non-modifiable risk factors for surgical site infection?  **Answer**  She believes that it would be useful to know about the non-modifiable risk factors as well as it can highlight that the patient may be at a higher baseline risk for infection. However, this can affect anxiety negatively in some patients. On the other hand it could also encourage proactivity, if the patient knows their baseline risk is higher, they can learn to look for early signs of infection and may be keen to keep a more accurate record. |
